# Infectious Virus Persists in CD4^+^ T Cells and Macrophages in Antiretroviral Therapy-Suppressed Simian Immunodeficiency Virus-Infected Macaques

**DOI:** 10.1128/JVI.00065-19

**Published:** 2019-07-17

**Authors:** Celina M. Abreu, Rebecca T. Veenhuis, Claudia R. Avalos, Shelby Graham, Suzanne E. Queen, Erin N. Shirk, Brandon T. Bullock, Ming Li, Kelly A. Metcalf Pate, Sarah E. Beck, Lisa M. Mangus, Joseph L. Mankowski, Janice E. Clements, Lucio Gama

**Affiliations:** a Department of Molecular and Comparative Pathobiology, Johns Hopkins University School of Medicine, Baltimore, Maryland, USA; b Department of Pathology, Johns Hopkins University School of Medicine, Baltimore, Maryland, USA; c Department of Neurology, Johns Hopkins University School of Medicine, Baltimore, Maryland, USA; d Vaccine Research Center, NIAID, NIH, Bethesda, Maryland, USA; Emory University

**Keywords:** CD4^+^ T cells, latency, macrophages, quantitative viral outgrowth assay, reservoir, simian immunodeficiency virus

## Abstract

Understanding the cellular and anatomical sites of latent virus that contribute to human immunodeficiency virus (HIV) rebound is essential for eradication. In HIV-positive patients, CD4^+^ T lymphocytes comprise a well-defined functional latent reservoir, defined as cells containing transcriptionally silent genomes able to produce infectious virus once reactivated. However, the persistence of infectious latent virus in CD4^+^ T cells in compartments other than blood and lymph nodes is unclear. Macrophages (Mϕ) are infected by HIV/simian immunodeficiency virus (SIV) and are likely to carry latent viral genomes during antiretroviral therapy (ART), contributing to the reservoir. Currently, the gold standard assay used to measure reservoirs containing replication-competent virus is the quantitative viral outgrowth assay (QVOA). Using an SIV-macaque model, the CD4^+^ T cell and Mϕ functional latent reservoirs were measured in various tissues using cell-specific QVOAs. Our results showed that blood, spleen, and lung in the majority of suppressed animals contain latently infected Mϕs. Surprisingly, the numbers of CD4^+^ T cells, monocytes, and Mϕs carrying infectious genomes in blood and spleen were at comparable frequencies (∼1 infected cell per million). We also demonstrate that *ex vivo* viruses produced in the Mϕ QVOA are capable of infecting activated CD4^+^ T cells. These results strongly suggest that latently infected tissue Mϕs can reestablish productive infection upon treatment interruption. This study provides the first comparison of CD4^+^ T cell and Mϕ functional reservoirs in a macaque model. It is the first confirmation of the persistence of latent genomes in monocytes in blood and Mϕs in the spleen and lung of SIV-infected ART-suppressed macaques. Our results demonstrate that transcriptionally silent genomes in Mϕs can contribute to viral rebound after ART interruption and should be considered in future HIV cure strategies.

**IMPORTANCE** This study suggests that CD4^+^ T cells found throughout tissues in the body can contain replication-competent SIV and contribute to rebound of the virus after treatment interruption. In addition, this study demonstrates that macrophages in tissues are another cellular reservoir for SIV and may contribute to viral rebound after treatment interruption. This new insight into the size and location of the SIV reservoir could have great implications for HIV-infected individuals and should be taken into consideration for the development of future HIV cure strategies.

## Abstract (precis)

This study suggests that CD4^+^ T cells found throughout tissues in the body can contain replication-competent SIV and contribute to rebound of the virus after treatment interruption. In addition, this study demonstrates that macrophages in tissues are another cellular reservoir for SIV and may contribute to viral rebound after treatment interruption. This new insight into the size and location of the SIV reservoir could have great implications for HIV-infected individuals and should be taken into consideration for the development of future HIV cure strategies.

## INTRODUCTION

The development of drugs that target multiple stages of the human immunodeficiency virus (HIV) life cycle and reduce viremia to undetectable levels have transformed HIV infection from a deadly disease to a manageable chronic illness (1–3). However, life-long antiretroviral therapy (ART) is necessary, since the regimen suppresses viral replication but does not eliminate the latent HIV reservoir (4–6), defined as cells that harbor integrated but transcriptionally silent HIV provirus that can be induced to produce infectious virus (7). Studies have demonstrated that multiple tissues harbor HIV provirus in ART-suppressed patients, suggesting that the latent HIV reservoir can be found throughout the body (8–14). Latently infected cells that harbor HIV serve as the source of viral rebound after ART interruption, and the half-life of latently infected cells precludes the elimination of the virus by ART alone (15). Therefore, understanding both the location and the types of cells that harbor latent HIV throughout the body is critical for HIV eradication.

HIV and the simian immunodeficiency virus (SIV) cause immunodeficiency disease as well as tissue-specific diseases in humans and macaques, respectively (16). CD4^+^ T cells isolated from peripheral blood and lymph nodes have been the most extensively characterized reservoirs due to the limitations associated with obtaining tissue samples from HIV-infected patients (9, 17–19). However, less than 2% of the total lymphocyte population resides in the peripheral blood, and CD4^+^ T cell populations are present in multiple tissues, potentially contributing to viral persistence (15). Additionally, not all CD4^+^ T cell populations contribute equally to the HIV reservoir; the central memory and transitional memory populations are thought to contain the majority of integrated HIV DNA compared to their effector memory counterpart (19, 20). SIV-infected macaques treated with ART have been used to examine the contribution of CD4^+^ T cells to the latent reservoir in other tissues (21–31). Additionally, this model provides the opportunity to study other cells that may also play a role in HIV persistence, such as monocytes, macrophages (Mϕ), astrocytes, and follicular cells (13, 15, 32). Tissue-resident Mϕs, such as splenic Mϕs, alveolar Mϕs, and microglia, are long-lived and relatively resistant to the cytopathic effects of HIV/SIV infection, potentially serving as stable viral reservoirs (33, 34).

Previous studies have assessed the frequency of HIV/SIV-infected cells in different tissues by quantifying HIV cell-associated RNA or DNA. These studies suggest that the presence of HIV DNA with little or no viral RNA is equivalent to latency (35–38). However, this approach overestimates the number of latently infected cells due to the presence of a large proportion of defective proviral DNA as well as intact genomes that do not express virus *in vivo* (39). To measure the functional latent reservoir, an assay that quantifies the number of latently infected resting CD4^+^ T cells, the quantitative viral outgrowth assay (QVOA), was developed and has been widely used to measure CD4^+^ T cell reservoirs in ART-suppressed HIV-infected patients (40, 41). We have adapted the HIV CD4^+^ T cell QVOA to be used in an SIV-infected macaque model (33) and also developed a QVOA for monocytes and tissue Mϕs (Mϕ QVOA) using the same SIV model (34). Using the Mϕ QVOA we have shown that monocytes from the blood and Mϕs from bronchoalveolar lavage fluid (BAL), lung, spleen, and brain of untreated SIV-infected macaques harbor replication-competent virus (33). Further, using this assay, we demonstrated that brain Mϕs constitute a functional latent reservoir in a model of ART-suppressed SIV-infected macaques (34).

To accelerate progress toward a cure, it is important to fully characterize the CD4^+^ T cell and Mϕ functional latent reservoirs throughout the body. In this study, we analyzed the reservoirs in spleen, lung, and blood of ART-suppressed SIV-infected macaques. We used the CD4^+^ T cell QVOA and Mϕ QVOA to determine the number of functionally latent cells in each compartment. Notably, latently infected CD4^+^ T cells, monocytes, and Mϕs were identified in all ART-suppressed macaques studied. This study and our previous findings demonstrate that CD4^+^ T cells and Mϕs represent functional latent reservoirs in many tissues.

## RESULTS

### Treatment regimen and characteristics of the SIV-pigtailed model.

**FIG 1:**
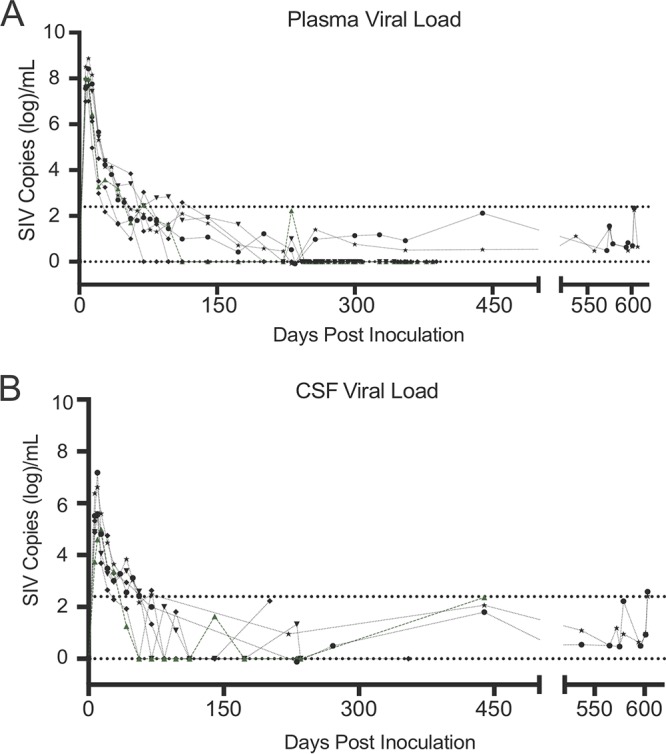
Viral load in plasma and CSF of seven suppressed SIV-infected ART-treated macaques (34). Seven SIV-infected pigtailed macaques were treated with similar ART regimens (tenofovir, darunavir, integrase inhibitor, and ritonavir). Symbols represent the plasma (A) and CSF (B) viral load values for each animal. Analyses of samples with values below the limit of quantitation (LOQ) for the SIV RT-qPCR assay (430 SIV RNA copies/ml; top dotted line) were repeated using RT-ddPCR (LOQ, 5 SIV RNA copies/ml; bottom dotted line).

Seven pigtailed macaques were inoculated with SIV/DeltaB670 and SIV/17E-Fr, an SIV model that uses macrophage-tropic viral strains to accurately reproduce the neuropathic and immunologic events identified in HIV-infected patients (42–45). Macaques were treated with ART at 12 days postinfection (dpi), when the reservoirs found in both the peripheral tissues and central nervous system (CNS) have been shown to be seeded in our model and, in the case of lymph nodes and peripheral blood mononuclear cells (PBMCs), in other SIV models as well (46, 47). All macaques were treated daily treatment with tenofovir (TFV), integrase inhibitor (INI), ritonavir (RTV), and darunavir (DRV) (Table 1). This ART regimen was chosen based on the CNS penetrance score (CPE) to fully suppress virus replication in both the CNS and the peripheral blood and tissues (48). The viral load in both plasma and cerebrospinal fluid (CSF) was measured longitudinally to demonstrate viral suppression in both the peripheral blood and the CNS (Fig. 1). Three of the seven suppressed animals (animals Pm12, Pm22, and Pm23) were also treated with latency-reversing agents (LRA) during ART suppression, although these treatments did not measurably alter the results reported here. All animals were suppressed (less than 50 copies per ml, as measured by digital droplet PCR [ddPCR]) for a minimum of 6 months and as long as 18 months in both CSF and plasma, as measured by an SIV *gag* RNA ddPCR. Two SIV-infected macaques, Pm26 and Pm27, the results for which are not depicted in the graphs, were used as untreated controls. Both of the animals had detectable plasma and CSF viral loads throughout the infection and at the terminal time point (Table 1).

**TABLE 1:** Characterization of SIV-infected ART-suppressed macaques at the terminal time point

Animal code^*a*^	ART duration (mo)	Cells count (no. of cells/μl blood)		Viral load (no. of SIV RNA copies/ml)		SIV infection status
		CD4^+^ cells	Monocytes	Plasma	CSF	
Pm11	20.5	474	1,092	<LOD^*b*^	<LOD	Suppressed
Pm12	20.5	1,074	485	<LOD	<LOD	Suppressed
Pm21	13.0	280	340	<LOD	<LOD	Suppressed
Pm22	13.0	324	2,310	<LOD	<LOD	Suppressed
Pm23	11.5	331	410	<LOD	<LOD	Suppressed
Pm24	6.0	291	350	<LOD	<LOD	Suppressed
Pm25	6.0	790	310	<LOD	<LOD	Suppressed
Pm26	0	481	710	11,993,458	15,364,703	Viremic
Pm27	0	436	580	13,610,950	2,935	Viremic

a Two viremic animals, Pm26 and Pm27, were used as positive controls in some experiments.

b <LOD, less than the limit of detection.

### SIV DNA but not RNA is detectable in whole tissue.

**FIG 2:**
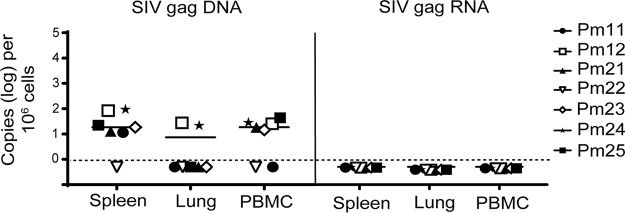
Quantification of SIV DNA and SIV RNA from tissue samples. Cellular DNA and RNA were extracted from spleen, lung, and PBMCs from seven SIV-infected ART-suppressed macaques. SIV *gag* DNA (left) and SIV *gag* RNA (right) were measured by ddPCR, with the limit of quantitation (LOQ, dashed lines) being 1 copy per reaction mixture. Open symbols indicate animals that were treated with LRAs *in vivo*; closed symbols indicate animals that were not treated with LRAs.

To evaluate whether the viral suppression observed in the plasma was reflected in PBMCs and tissues, such as spleen and lung, the levels of SIV *gag* DNA and RNA were measured in these compartments using reverse transcriptase (RT) quantitative PCR (qPCR) and RT-ddPCR. Of the seven ART-treated macaques studied, SIV *gag* DNA was detected in the spleens of six macaques, in the PBMCs of five macaques, and in the lungs of two macaques. Pm12 and Pm24 had detectable SIV DNA in all three compartments, with the highest level of DNA being in the spleen (Fig. 2, left). Pm21, Pm23, and Pm25 had similar levels of detectable SIV DNA in both the spleen and PBMCs, whereas Pm11 had detectable SIV DNA only in the spleen. Pm22 was undetectable in all three compartments. No correlation was observed between the amount of DNA detected in the tissues and the length of treatment. All seven ART-treated macaques had undetectable levels of SIV *gag* RNA in the PBMCs, spleens, and lungs, as measured by RT-ddPCR at the terminal time point (Fig. 2, right).

### SIV DNA is detectable in monocytes/Mϕs and CD4^+^ T cells isolated from blood and tissue.

**FIG 3:**
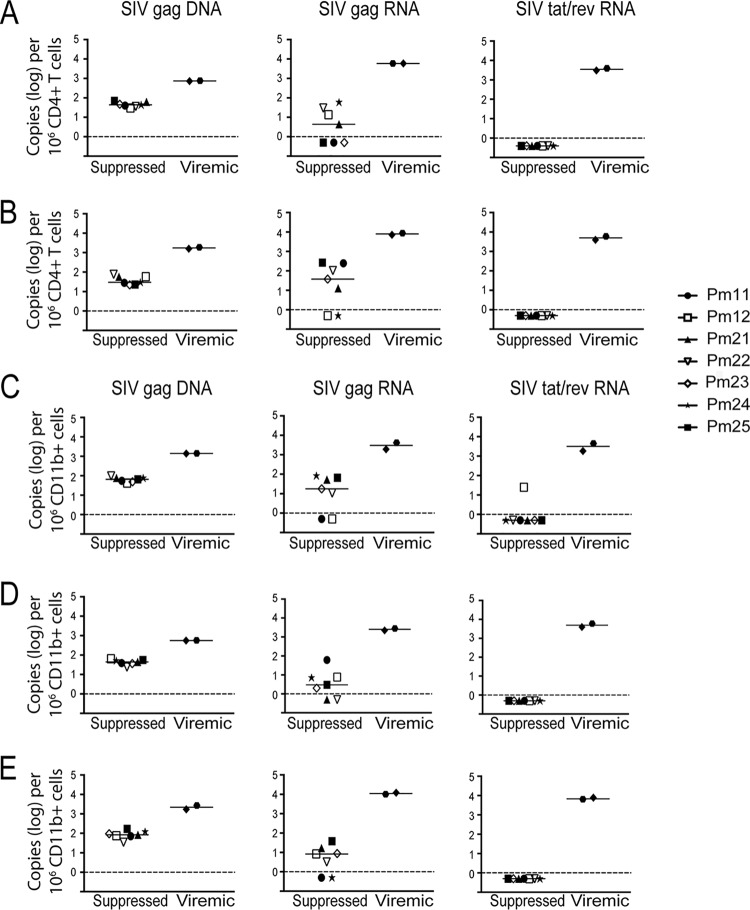
Quantification of SIV DNA and RNA from CD4^+^ T cells and CD11b^+^ cells isolated from tissues. CD4^+^ T cells (A and B) and CD11b^+^ cells (C, D, and E) were isolated from spleen, lung, and PBMCs from seven SIV-infected ART-suppressed macaques and two SIV-infected untreated macaques. Cellular DNA and RNA were then extracted and analyzed for SIV *gag* DNA (left), SIV *gag* RNA (middle), and SIV *tat*/*rev* RNA (right) by PCR. The limit of quantitation (LOQ) for ddPCR is 1 copy per reaction, and that for qPCR is 10 copies per reaction. The dashed lines represent the LOQ for ddPCR. Open symbols indicate animals that were treated with LRAs *in vivo*; closed symbols indicate animals that were not treated with LRAs.

**FIG 4:**
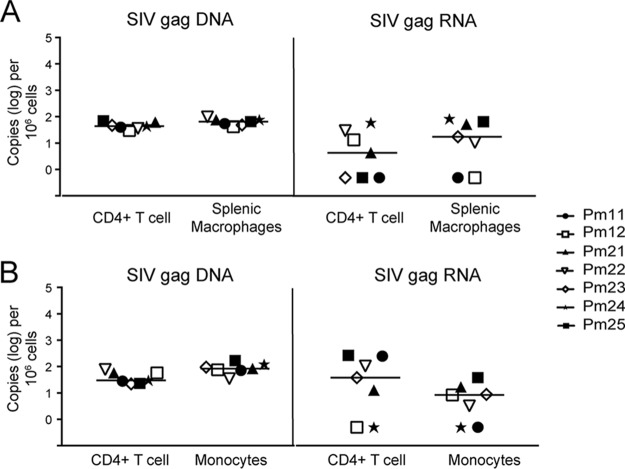
SIV DNA and RNA levels were similar between CD4^+^ T cells and macrophages or monocytes isolated from tissues. Comparison of SIV DNA and SIV RNA levels in CD4^+^ T cells and CD11b^+^ cells isolated from spleen (A) and blood (B). The dotted line represents the limit of quantitation for ddPCR. Each symbol represents an animal. Open symbols indicate animals that were treated with LRAs *in vivo*; closed symbols indicate animals that were not treated with LRAs.

**FIG 5:**
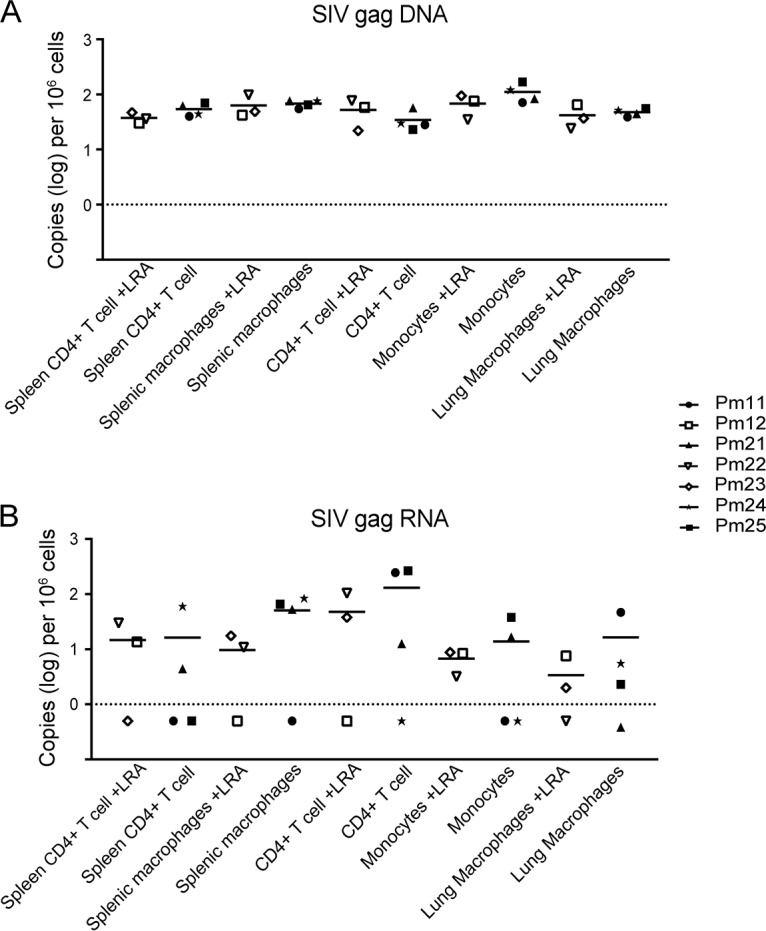
*In vivo* LRA treatment does not have a measurable effect on the level of SIV *gag* DNA or RNA in isolated cells. SIV *gag* DNA (A) and RNA (B) levels measured in cells isolated from SIV-infected macaques treated with LRA were compared to those measured in cells isolated from SIV-infected macaques without LRA treatment. CD4^+^ T cells were isolated from blood and spleen. CD11b^+^ cells (monocytes/macrophages) were isolated from blood, spleen, and lung.

We hypothesized that SIV DNA is not measurable in the spleen, lung, and PBMCs of some animals due to the limit of quantitation for the RT-qPCR assay used to evaluate homogenized tissue (10 copies/μg total DNA or RNA). Therefore, to increase the sensitivity, we evaluated SIV DNA and RNA by ddPCR in enriched CD11b^+^ (monocytes/Mϕs) and CD4^+^ T cells isolated from these tissues and blood. CD11b^+^ cells were isolated from all three compartments, whereas CD4^+^ T cells, whose numbers are very low in the lung, were isolated from the spleen and blood. In contrast to whole tissue, all monocytes/Mϕs and CD4^+^ T cell isolations from the spleen, lung, and blood of the suppressed animals were positive for SIV DNA, with levels ranging from 23 to 168 copies per 10^6^ cells (Fig. 3A to E). The viremic animals had detectable SIV DNA in all samples, with the levels ranging from 1,392 to 1,424 copies per 10^6^ cells. Notably, the amounts of SIV DNA were similar between CD4^+^ T cells and monocytes/Mϕs isolated from spleen and blood (Fig. 4A and B). The length of ART treatment and LRA treatment and the duration of viral suppression did not appear to influence the levels of SIV DNA in either cell type (Fig. 5A).

### Presence of residual SIV *gag* but not *tat-rev* multiply spliced RNA in monocytes/Mϕs and CD4^+^ T cells isolated from tissues and blood.

To evaluate transcriptional activity in monocytes/Mϕs and CD4^+^ T cells isolated from tissues, SIV cell-associated RNA (caRNA) was quantitated by RT-ddPCR and RT-qPCR. caRNA was isolated from the same samples from which DNA was isolated, as described above, and used for quantification of unspliced SIV *gag* RNA and multiply spliced SIV *tat/rev* RNA. SIV *gag* RNA was detectable in the spleen CD4^+^ T cells of four suppressed macaques and in the circulating CD4^+^ T cells of five suppressed macaques (Fig. 3A and B). In addition, SIV *gag* RNA was detectable in CD11b^+^ cells isolated from the spleen, lung, and blood of five suppressed macaques (Fig. 3C to E). Notably, the levels of SIV *gag* RNA were similar in both the CD11b^+^ and CD4^+^ T cells isolated from each comparable compartment (Fig. 4A and B). As a control, SIV *gag* RNA was also assessed in viremic macaques in CD11b^+^ and CD4^+^ T cells from the same compartments. The SIV *gag* RNA level was 100-fold higher in all cells isolated from viremic animals than in all cells isolated from suppressed animals (Fig. 3). Similar to SIV DNA levels, the length of ART treatment and LRA treatment and the duration of viral suppression did not appear to influence the levels of SIV RNA in either cell type (Fig. 5B).

Detection of SIV *gag* RNA does not necessarily indicate active transcription, nor does the presence of SIV *tat/rev* RNA (49, 50). Therefore, we also measured the presence of SIV *tat/rev* multiply spliced RNA in all isolated cells. All CD4^+^ T cell samples isolated from the spleen and blood of suppressed macaques were negative for SIV *tat/rev* RNA (Fig. 3A and B). In addition, all but one CD11b^+^ samples isolated from the spleen, lung, and blood were negative for SIV *tat/rev* RNA (Fig. 3C to E). The CD11b^+^ spleen sample (Pm12) with detectable SIV *tat/rev* RNA had 25 copies per 10^6^ cells, which is close to the limit of quantitation for the assay (Fig. 3C) and substantially lower than the levels from any of the viremic animals (Fig. 3A to E). Although it is known that multiply spliced viral RNAs are usually detected at lower levels than the unspliced forms (51), these results suggest that the residual SIV *gag* RNA detected in the isolated CD11b^+^ cells (monocytes/Mϕs) and CD4^+^ T cells in suppressed macaques is not indicative of active replication but indicates expected stochastic oscillations (52). Therefore, detection of virus in the QVOA would suggest reactivation of latent viral genomes.

### Macrophage numbers and SIV DNA levels are stable over time.

**FIG 6:**
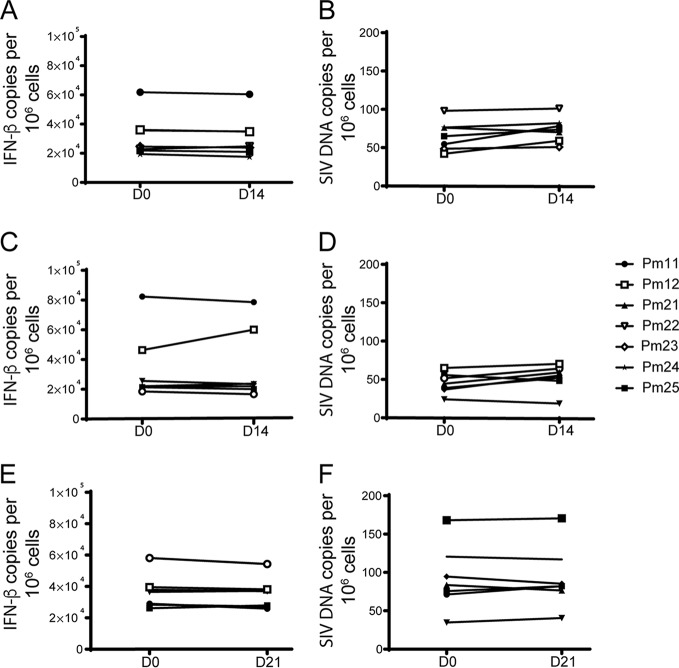
The macrophage population in culture is stable over time. IFN-β gene (A, C, and E) and SIV *gag* (B, D, and F) DNA copies were quantitated by qPCR in CD11b^+^ cells isolated from spleen, lung, and PBMCs before being plated for Mϕ QVOA and also from cells in the QVOA wells at 14 or 21 days postplating. Values are presented as the number of copies per 10^6^ cells. Open symbols indicate animals that were treated with LRAs *in vivo*; closed symbols indicate animals that were not treated with LRAs. D0, D14, and D21, days 0, 14, and 21, respectively.

Mϕs, unlike CD4^+^ T cells, do not proliferate *in vitro*. Instead, the number of Mϕs remains stable and viable in culture for up to 3 weeks (33). To demonstrate that there was no expansion of the Mϕs derived from spleen, lung, and blood, beta interferon (IFN-β) gene expression was measured in Mϕs at days 0 and 14 or 21 in culture, and the number of cells in each well remained stable (Fig. 6A, C, and E). The IFN-β gene is a single-copy gene and is used to quantify the number of cells present in our assays. In parallel, SIV DNA was measured at days 0 and 14 or 21, and there was no significant change in the level of SIV DNA over time (Fig. 6B, D and F). These data corroborate that, unlike CD4^+^ T cells in culture, Mϕs do not proliferate and are resistant to cell death, confirming that these long-lived cells could constitute a viable reservoir *in vivo*.

### Functional latent virus is present in CD4^+^ T cells and Mϕs in the blood and spleen of suppressed SIV-infected macaques.

**FIG 7:**
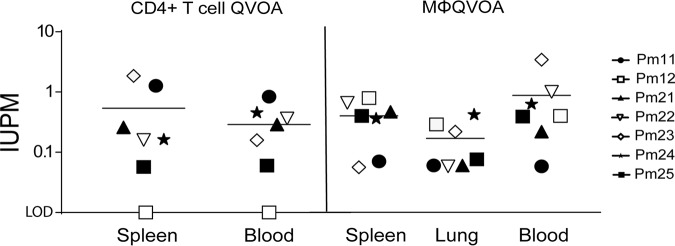
Functional latent reservoirs are detected in both CD4^+^ T cells and monocytes/macrophages isolated from SIV-infected ART-suppressed animal tissues. The frequency of latently infected CD4^+^ T cells (left) and monocytes/macrophages (right) isolated from the spleen, lung, and PBMCs of suppressed ART-treated macaques were quantitated by cell-specific QVOAs. The horizontal black lines represent the median number of infectious units per million cells (IUPM), and each symbol represents an animal. Open symbols indicate animals that were treated with LRAs *in vivo*; closed symbols indicate animals that were not treated with LRAs. LOD, limit of detection.

**FIG 8:**
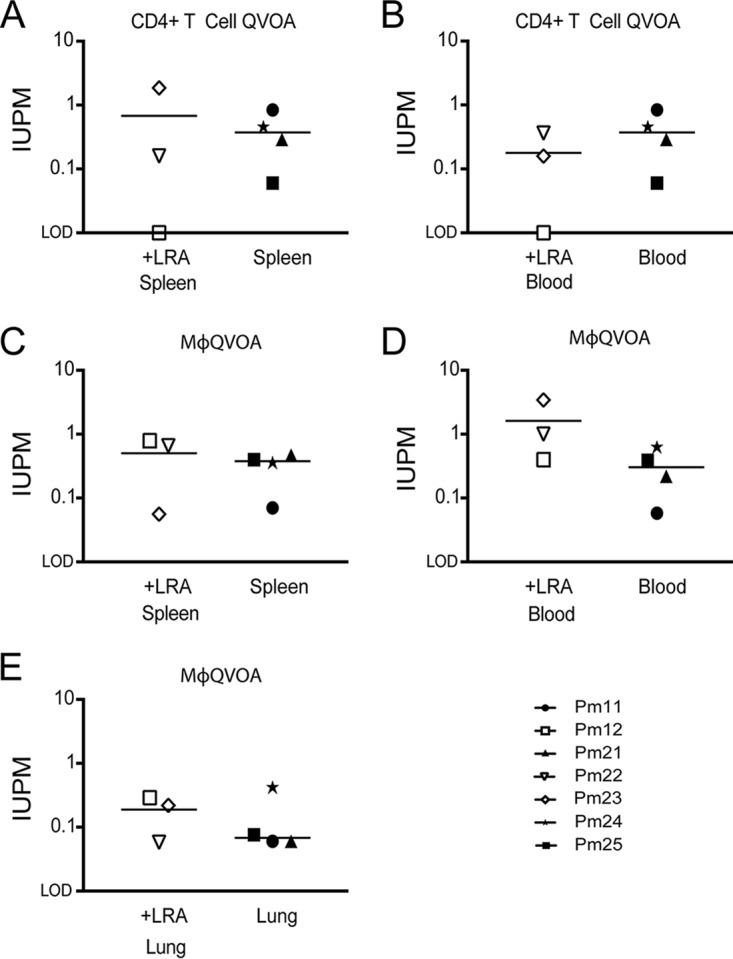
*In vivo* LRA treatment has no measurable effect on IUPM. A comparison of the IUPM values obtained from cells isolated from SIV-infected macaques treated with LRA compared to those obtained from cells isolated from SIV-infected macaques without LRA treatment is shown. (A) Number of IUPM from CD4^+^ T cells isolated from spleen; (B) number of IUPM from CD4^+^ T cells isolated from blood; (C) number of IUPM from macrophages isolated from spleen; (D) number of IUPM from monocyte-derived macrophages isolated from blood; (E) number of IUPM from alveolar macrophages isolated from lung.

CD4^+^ T cells have been extensively characterized as the primary latent reservoir in peripheral blood and lymph nodes in ART-suppressed HIV-positive (HIV^+^) patients and ART-suppressed SIV-infected animals in our previous studies (17, 53–56). Previously, our studies showed that Mϕs also contribute to the reservoir in the brains of suppressed SIV-infected animals (34). We hypothesized that the spleen, an additional site that harbors large numbers of Mϕs and CD4^+^ T cells, also contributes to the SIV reservoir and may act as a sanctuary for latently infected cells. To estimate the frequency of latently infected CD4^+^ T cells and Mϕs in the spleen, we isolated both CD4^+^ lymphocytes and Mϕs from the spleen and used the quantitative viral outgrowth viral assays (QVOA) specific for each of these cells (33). Six of the seven suppressed macaques had quantifiable levels of latently infected CD4^+^ T cells in both the spleen and blood (Fig. 7 and Table 2). One suppressed animal, Pm12, had undetectable numbers of infectious units per million (IUPM) in the CD4 QVOA, which can be explained by the limited number of cells used for the assay compared to the number in the other samples (Fig. 7 and Table 2). All Mϕ QVOAs from the spleen and blood of the seven ART-suppressed macaques had measurable IUPM values (Fig. 7 and Table 2). The median Mϕ IUPM values from blood and spleen were similar, as were the median CD4 IUPM values from the blood and spleen, suggesting that the level in the blood may be a surrogate representation for the level of persistent virus in the spleen. Additionally, the median CD4 IUPM values and median Mϕ IUPM values from the same tissues were similar, regardless of the duration of ART treatment or cell-associated SIV *gag* DNA and RNA levels (Table 1 and Fig. 3), suggesting that both cell types have the potential to contribute equally to the SIV reservoir. Taken together, these data suggest that viral latency in the spleen and blood is maintained despite undetectable levels of viremia in the plasma and CSF for an extended time in the SIV-macaque model. Additionally, as seen with SIV DNA and RNA levels, the length of ART and LRA treatment and the duration of viral suppression did not appear to influence IUPM levels in either cell type (Fig. 8).

**TABLE 2:** Quantitation of IUPM in CD4^+^ T cell and monocyte/macrophage QVOA

Animal code	IUPM^*a*^				
	CD4^+^ T cell QVOA		Monocyte/macrophage QVOA		
	Spleen	Blood	Spleen	Lung	Blood
Pm11	1.27	0.84	0.07	0.06	0.06
Pm12	<LOD	<LOD	0.79	0.29	0.40
Pm21	0.26	0.29	0.47	0.06	0.22
Pm22	0.16	0.37	0.66	0.06	1.00
Pm23	1.86	0.16	0.06	0.22	3.44
Pm24	0.16	0.46	0.36	0.42	0.63
Pm25	0.06	0.06	0.40	0.08	0.39

a IUPM was quantitated as described by Avalos et al. (33). IUPM values represent the presence or absence of positive wells with more than 50 SIV *gag* RNA copies/ml of supernatant as determined by RT-qPCR, as described in the legend to Fig. 5. Values reflect maximum likelihood estimates of the infection frequency (in numbers of IUPM). Given the resolution of the assay, the 95% confidence interval was typically 0.2 to 4 times the reported value. <LOD, less than the limit of detection.

### Viral persistence in alveolar Mϕs in suppressed SIV-infected macaques.

Mϕs are present in every tissue in the body, suggesting that latent HIV/SIV could be detected in other organs. We examined alveolar Mϕs to determine whether they also harbored SIV DNA and whether these lung Mϕs could contribute to the SIV reservoir (Fig. 3D). All CD11b^+^ cells isolated from the lungs of suppressed macaques produced detectable and quantifiable levels of SIV in the QVOA (Fig. 7 and Table 2). Similar to the spleen and blood QVOAs, treatment duration, LRA treatment, and length of suppression did not correlate with IUPM values. These studies demonstrate that Mϕs in the lung and spleen are latently infected in ART-suppressed macaques and have the potential to contribute to the latent reservoir.

### Reactivated latent viruses released from Mϕs are replication competent.

**FIG 9:**
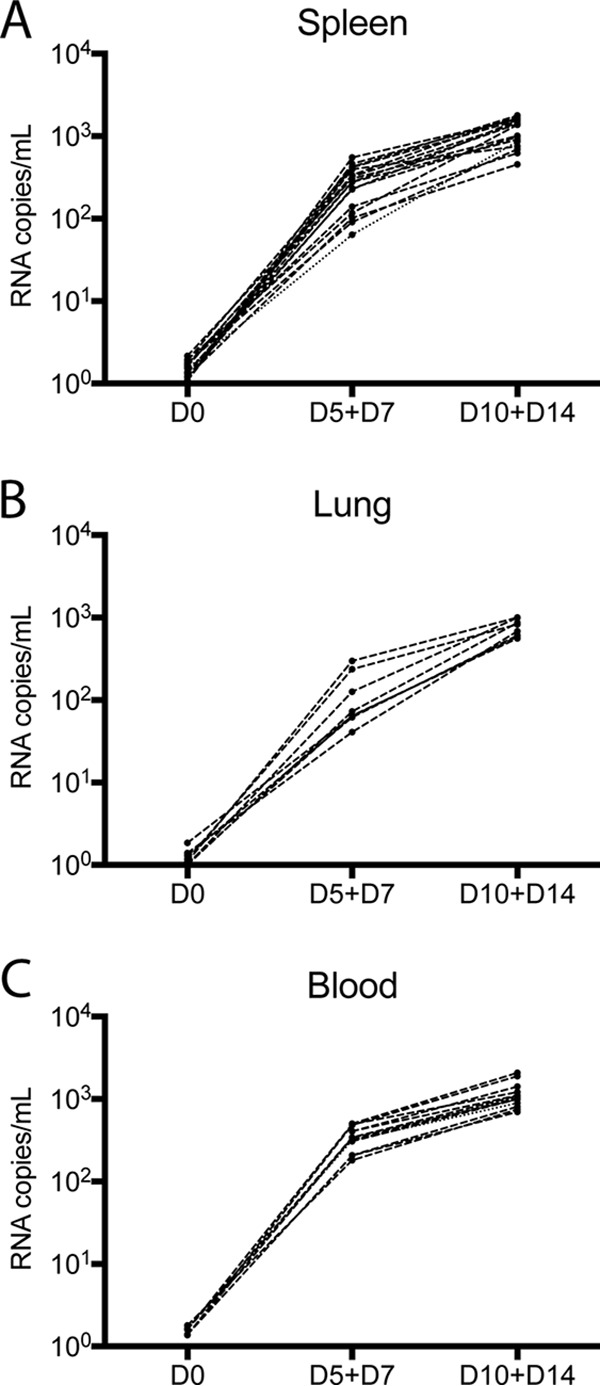
Supernatants from Mϕ QVOA cultures contain viruses that establish *de novo* infection in activated macaque PBMCs. Activated PBMCs from a healthy pigtailed macaque were spinoculated with culture supernatant from positive Mϕ QVOA wells with spleen tissue (A), lung tissue (B), and blood (C) from the seven suppressed macaques. The graphs show the viral kinetics over time postinfection, as measured by an SIV RNA RT-qPCR. Each line represents viral replication from one Mϕ QVOA well.

To confirm that the SIV RNA measured in the QVOA from spleen, lung, and blood Mϕs reflected infectious virus capable of *de novo* infection in cells, supernatants from Mϕ QVOA wells were used to infect activated healthy PBMCs from macaques. Virus derived from the Mϕ QVOA expanded exponentially in PBMCs by day 5 and continued to expand through day 14 (Fig. 9). These results demonstrate that virus derived from latently infected Mϕs in both blood and tissues is infectious and can contribute to the viral rebound once ART is interrupted.

## DISCUSSION

Despite continued improvements in antiretroviral therapy, HIV persists in millions of individuals worldwide. The greatest barrier to an HIV cure is the functional latent reservoir. Therefore, it is essential to understand the cell types that contribute to the latent reservoir, the anatomical locations, and the potential of these cells to contribute to viral resurgence after ART interruption. HIV cure strategies are aimed at either eliminating the reservoir, maintaining latency by immunologic control, or other approaches to suppress viral transcription (57, 58). The majority of previous studies have focused on latent HIV/SIV in CD4^+^ T cells in the tissues of ART-suppressed patients and animal models but have limited their studies to lymph nodes and blood, with little focus on other tissues, such as the spleen and lung (27, 49, 59). Monocytes and tissue-specific Mϕs have not been considered to contribute to the functional reservoir. The primary goal of this study was to examine the role of Mϕs as functional latent reservoirs in SIV-infected ART-suppressed macaques.

Current ART regimens used to treat HIV-infected patients and used in SIV-macaque studies are highly effective in reducing viremia to undetectable levels (21, 29). In this study, the viral loads in the plasma and CSF were measured longitudinally in seven SIV-infected pigtailed macaques, demonstrating that virus replication was controlled in both the periphery and the CNS. Plasma viral loads are easily accessible in HIV^+^ patients; however, whether the plasma viral load reflects the level of HIV in tissues is unknown. To address this question, we assessed SIV DNA and RNA levels in the PBMCs, spleens, and lungs of SIV-infected ART-suppressed macaques. All CD4^+^ T cells and myeloid cells isolated from tissues had detectable levels of SIV *gag* DNA (Fig. 2). Interestingly, no correlation was observed between the amount of DNA detected in the tissues and the length of treatment (data not shown). This may have been due to the short length of time that the macaques were suppressed compared to that for HIV-infected individuals (60). However, Whitney and colleagues showed that only SIVmac251-infected macaques started early on ART treatment (day 3 postinfection) and not macaques started on treatment at later time points (days 7 to 14) decreased the viral RNA and proviral DNA levels in peripheral blood, lymph nodes, and gastrointestinal mucosa (47). Additionally, the *in vivo* LRA treatments in three animals did not affect SIV DNA levels in the cells isolated from tissues (Fig. 5A). In contrast, all CD4^+^ T cells and most myeloid cells isolated from PBMCs, spleen, and lung had undetectable levels of SIV *tat*/*rev* RNA, and some had detectable *gag* RNA (Fig. 5B). Previous studies using cells from HIV-suppressed patients have shown that the predominant cellular HIV type 1 (HIV-1) RNA species detected during ART suppression is HIV *gag*, whereas HIV-1 *tat*/*rev* was found to be 10 to 100 times less abundant (61–63). The demonstration that SIV *tat*/*rev* RNA was undetectable, despite detectable levels of SIV *gag* DNA and RNA, supports the conclusion that SIV expression was transcriptionally silent in the tissues of these animals.

The presence of HIV DNA is not an accurate representation of the level of intact and activatable virus within tissues, since defective proviruses tend to outnumber intact proviruses 20 to 1 (64). In this study, we have shown that CD4^+^ T cells and myeloid cells isolated *ex vivo* can be reactivated to produce infectious virus in culture. These findings are novel and demonstrate that the Mϕ reservoir is not limited to the brain but that monocytes in blood and Mϕs in spleen and lung also harbor latent SIV genomes that, upon reactivation, can produce infectious virus *ex vivo*. Monocytes play an important role in immune surveillance and can enter tissues and mature into macrophages. The short half-life in monocytes suggests that these cells cannot represent a viral reservoir. However, entry into tissues of latently infected monocytes that mature into long-lived macrophages would constitute a reservoir. To determine the stability of the Mϕ reservoir, we assessed cell longevity and DNA stability in long-term *ex vivo* cultures. The CD4^+^ T cell reservoir is likely maintained by lymphocyte proliferation and the division of latently infected cells (55, 65–67). Similarly, macrophages directly originated from the yolk sac, such as red pulp and alveolar macrophages, may maintain their populations through cell division and self-renewal (68). In this study, we have demonstrated that *ex vivo* Mϕs are long-lived and maintain stable levels of proviral DNA in culture for at least 21 days (Fig. 6). Other groups have demonstrated that monocyte-derived macrophages with a shorter life span, such as interstitial macrophages in the lung, can also be infected (69), although the role that they play in the maintenance of the reservoir is still unexplored. Additionally, the viruses released in the Mϕ QVOAs are replication competent and infect PBMCs from healthy macaques (Fig. 9). These data suggest that although Mϕs do not release virus as robustly as CD4^+^ T cells, the virus that is released is infectious and capable of reestablishing infection after ART interruption.

The mechanism that establishes latency in macrophages is unclear but would be expected to be different from that in CD4^+^ T cells. The mechanisms by which CD4^+^ T cell establish latency are well-known and consist of two different processes. First, naive T cells carry the HIV-1 unintegrated DNA preintegration complex; therefore, naive T cells in preintegration latency are short-lived and may not contribute to long-term HIV-1 persistence. Second, memory T cells carry HIV-1 proviral DNA into the host’s genome postintegration and play an important role in long-term latency (70, 71). However, Kelly and colleagues have shown in an *in vitro* examination of HIV infection of macrophages that unintegrated viral DNA not only has an unusual stability but also maintains biological activity and may provide a mechanism of latency in macrophages (72).

The presence of persistent infectious virus in myeloid cells during ART remains controversial in HIV infection. Many HIV-1 strains replicate efficiently in macrophages independently of the presence of Vpx (73, 74), an auxiliary protein present in HIV-2 and SIV that specifically enhances viral replication in macrophages (75, 76). Comparably, the Vpr present in HIV-1 recruits UNG2 into virions and modulates viral mutation rates in macrophages (77–79), indicating that these long-lived cells may carry intact genomes *in vivo*. A recent study reported that resident urethral Mϕs isolated from penile tissues contained latent HIV and could be reactivated *ex vivo* (80). However, previous work suggested that though liver Mϕs contained HIV DNA, the cells could not be reactivated *ex vivo* and infect activating CD4 T cells, suggesting that the integrated DNA was not functional (81). These studies point to the possibility that functional Mϕ reservoirs may not be present in all tissues but also highlight the difficulty of working with macrophages isolated from tissue. A 2014 study of a Mississippi baby suggested that myeloid cells may contribute to viral persistence, since there was no detectable viral DNA in resting CD4^+^ T cells but HIV DNA was present in monocyte-derived adherent cells (82). At 2 years after treatment interruption, the child rebounded and was immediately placed on ART. In addition, two HIV^+^ individuals with lymphoma who received allogeneic hematopoietic stem cell transplants (HSCT) had undetectable HIV RNA by standard clinical assays. However, at 3 and 8 months after ART interruption, both patients experienced a spontaneous and rapid rebound of HIV RNA in the plasma and CSF, accompanied by neurological symptoms (83). These data suggest that HIV may rebound from alternative reservoirs found in monocytes and Mϕs, which are not typically assessed prior to or during treatment interruption. Despite the limited numbers of animals analyzed and the broad probability range associated with QVOAs, our study provides new insight into the size and location of the SIV reservoir within tissues as well as provides evidence that SIV may rebound from CD4^+^ T cells and Mϕs after ART interruption and that both should be considered in the development of future HIV cure strategies.

## MATERIALS AND METHODS

### Ethics statement.

Animal studies were conducted in accordance with the guidelines of the Weatherall report (84), the *Guide for the Care and Use of Laboratory Animals* (85), and the USDA Animal Welfare Act. All studies were approved by the Johns Hopkins University Institutional Animal Care and Use Committee under protocol PR12M310. Animals were monitored twice daily by trained technicians or veterinarians for clinical signs of disease, such as weight loss, intractable diarrhea, and opportunistic infection, so that an early endpoint could be performed if necessary. Macaques were housed in Johns Hopkins University facilities, which are fully accredited by the Association for the Assessment and Accreditation of Laboratory Animal Care International (AAALAC), and fed a balanced Purina Mills macaque chow (Gray Summit, MO, USA). All animals were housed in groups prior to infection and in cages providing 6 square feet of space with environmental enrichment (manipulanda and novel foodstuffs), as well as visual and auditory contact with conspecifics. As mandated by the USDA, animals were housed in pairs following infection, which helped ensure that the animals received the appropriate drug dosage.

### Animal studies.

Seven juvenile male pigtailed macaques (Macaca nemestrina) were inoculated intravenously with the SIV/DeltaB670 swarm and the molecular clone SIV/17E-Fr as previously described (42–44). Macaques were treated at 12 days postinoculation (dpi) with 30 mg/kg of body weight of tenofovir (Gilead, Foster City, CA) for the first 2 weeks and then with 10 mg/kg of tenofovir once daily intramuscularly as previously described (34), as well as with 480 mg/kg darunavir (Janssen, Titusville, NJ), 10 mg/kg of integrase inhibitor L000870812 (Merck, Kenilworth, NJ), and 24 mg/kg ritonavir (AbbVie, North Chicago, IL); the last three drugs were administered orally twice daily. In addition to antiretroviral drugs, Pm12, Pm22, and Pm23 were treated with latency-reversing agents (LRA) after 550, 242, and 249 dpi, respectively (86). The LRA regimen consisted of the protein kinase C activator ingenol-B (Amazônia Fitomedicamentos Ltda., Brazil) and the histone deacetylase inhibitor vorinostat (suberoylanilide hydroxamic acid [SAHA]; Merck). Blood and CSF were drawn from each animal monthly prior to necropsy. Viral loads in plasma and CSF as well as absolute CD4^+^ T cell and monocyte counts are reported in Table 1. These studies were performed in accordance with federal guidelines and institutional policies. All manipulations were done while the animals were anesthetized with ketamine-HCl (Parke-Davis). Macaques were euthanatized at 194 to 628 days dpi using sodium pentobarbital while the animals were under ketamine sedation (10-mg/kg intramuscular injection) prior to perfusion with phosphate-buffered saline (PBS) to remove blood from tissues as previously described (43).

### Isolation of myeloid cells and lymphocytes from blood and tissue.

Peripheral blood mononuclear cells (PBMCs) were isolated by density gradient centrifugation on a 1.077-g/ml Percoll/Hanks gradient (GE Healthcare, Pittsburgh, PA) according to the manufacturer’s protocol. Spleen and lung cells were mechanically removed from tissues using an 18-gauge needle and passed through a 100-μm-mesh-size cell strainer. Blood, lung, and spleen Mϕs were cultured in Dulbecco modified Eagle medium (Life Technologies) supplemented with 20% heat-inactivated human type AB serum (Gemini Bio Products, West Sacramento, CA), 100 U/ml penicillin-streptomycin (Life Technologies), 20 μg/ml gentamicin (Life Technologies), 2 mM l-glutamine (Life Technologies), 2 mM sodium pyruvate (Sigma), 10 mM HEPES buffer (Life Technologies), and 50 ng/ml recombinant human Mϕ colony-stimulating factor (MCSF; R&D, Minneapolis, MN). CD4^+^ T cells were cultured in RPMI 1640 medium supplemented with 10% heat-inactivated bovine serum (Atlanta Biologicals), 100 U/ml penicillin-streptomycin (Life Technologies), 1% T cell growth factor, and 100 U/ml interleukin-2 (IL-2; Novartis, New York, NY).

### Spleen, lung, and PBMC monocyte/Mϕ QVOAs.

Mϕ quantitative viral outgrowth assay (QVOAs) were completed on myeloid cells isolated from PBMCs, spleen, and lung as previously described (33). In brief, myeloid cells were purified using nonhuman primate CD11b^+^ MicroBeads (Miltenyi Biotec, Auburn, CA) according to the manufacturer’s recommendation. Plates were coated with poly-l-lysine solution (Sigma) for at least 30 min and washed twice with PBS prior to cell plating. Purified Mϕs were cultured in duplicate in a 10-fold limiting dilution in the presence of 10 μM zidovudine (Sigma), 5 nM raltegravir (Merck), and 25 nM darunavir (Janssen, Titusville, NJ). Mϕs isolated from the spleen and lung were incubated for 2 to 4 days to allow for adherence. Monocytes isolated from PBMCs were incubated for 7 days to allow for Mϕ differentiation. Cells were then rinsed once with PBS to remove nonadherent cells and replenished with activation medium containing 10 ng/ml tumor necrosis factor (TNF; ProSpec), 1 μg/ml Pam3CSK4 (Sigma), and 1 μg/ml prostaglandin E_2_ (Sigma). Between 10^6^ and 10^4^ CEMx174 cells were added to all wells except those with T cell receptor β (TCRβ) controls, as previously described (33). Supernatants were collected and replenished with TNF, Pam3CSK4, and prostaglandin E_2_ every 2 days and assessed for SIV RNA by RT-qPCR. Supernatants from early time points (days 2, 4, 6) and later time points (days 8, 10, 12) were each pooled and assessed for viral RNA as described below. Cells were collected at day 14 and lysed in Buffer RLT Plus (Qiagen) with β-mercaptoethanol for RNA and DNA isolation. The frequency of cells harboring replication-competent virus was determined using the IUPMStats (v1.0) infection frequency calculator and expressed as the number of infectious units per million (IUPM) (40, 41). All Mϕ QVOAs were assessed for CD3^+^ T cell contamination using RT-qPCR for TCRβ as previously described (Table 3) (33). The total number of monocytes/macrophages assessed in the Mϕ QVOAs is described in Table 4.

**TABLE 3:** Frequency of infected CD4^+^ T cells among macrophages isolated from spleen, lung, and blood Mϕ QVOAs

Compartment and animal code	No. of CD4*^+^* T cell IUPM^*a*^	No. of CD3^+^ T cells in Mϕ QVOA well	% CD3^+^ CD4^+^ CD8^−^ T cells in blood at necropsy	No. of CD4^+^ T cells in Mϕ QVOA well	Probability of an infected CD4^+^ T cell present in Mϕ QVOA well	% chance an infected CD4^+^ T cell is present in an Mϕ QVOA well	IUPM Mϕ QVOA
Spleen							
Pm11	1.27	0.88	50.9	0.45	5.69E−07	0.00	0.07
Pm12	0.00	0.53	60.2	0.32	0.00	0.00	0.79
Pm21	0.26	0.56	55.2	0.31	0.00	0.00	0.47
Pm22	0.16	0.46	56.8	0.26	0.00	0.00	0.66
Pm23	1.86	0.12	56.4	0.07	0.00	0.00	0.06
Pm24	0.16	0.52	45.8	0.24	0.00	0.00	0.36
Pm25	0.06	0.54	48.9	0.26	0.00	0.00	0.40
Lung^*a*^							
Pm11	0.84	0.37	50.9	0.19	0.00	0.00	0.06
Pm12	0.00	0.40	60.2	0.24		0.00	0.29
Pm21	0.29	2.40	55.2	1.32	0.00	0.00	0.06
Pm22	0.37	0.36	56.8	0.20	0.00	0.00	0.06
Pm23	0.16	0.46	56.4	0.26	0.00	0.00	0.22
Pm24	0.46	0.58	45.8	0.27	0.00	0.00	0.42
Pm25	0.06	0.28	48.9	0.14	0.00	0.00	0.08
PBMC							
Pm11	0.84	0.49	50.9	0.25	0.00	0.00	0.06
Pm12	0.00	0.62	60.2	0.37		0.00	0.40
Pm21	0.29	0.64	55.2	0.35	0.00	0.00	0.22
Pm22	0.37	0.19	56.8	0.11	0.00	0.00	1.00
Pm23	0.16	0.15	56.4	0.09	0.00	0.00	3.44
Pm24	0.46	0.36	45.8	0.16	0.00	0.00	0.63
Pm25	0.06	0.15	48.9	0.07	0.00	0.00	0.39

a CD4^+^ T cell IUPM values were based on blood QVOA results.

**TABLE 4:** Total number of cells assessed in each QVOA

Animal code	Total no. of cells					
	Spleen QVOA		Lung QVOA		PBMC QVOA	
	Mϕ	CD4^+^ T cells	Mϕ	CD4^+^ T cells	Mϕ	CD4^+^ T cells
Pm11	6.44E + 07	4.37E + 06	1.78E + 07	ND^*a*^	1.27E + 07	2.50E + 07
Pm12	2.22E + 06	5.00E + 05	2.22E + 07	ND	2.22E + 07	5.00E + 06
Pm21	1.38E + 07	4.50E + 07	1.11E + 07	ND	1.93E + 07	5.50E + 07
Pm22	7.78E + 07	4.37E + 06	3.80E + 06	ND	5.11E + 06	2.50E + 07
Pm23	7.78E + 07	4.37E + 06	3.80E + 06	ND	5.11E + 06	2.50E + 07
Pm24	2.89E + 07	2.50E + 07	2.89E + 06	ND	6.67E + 06	2.50E + 07
Pm25	4.07E + 07	2.50E + 07	1.30E + 07	ND	3.82E + 06	2.50E + 07

a ND, not done.

### CD4^+^ T cell QVOA.

CD4^+^ T cells were isolated from the remaining cells after CD11b^+^ selection, and the levels of latently infected CD4^+^ T cells in spleen and blood were assessed using the CD4^+^ T cell QVOA as previously described (33, 87). The total numbers of CD4^+^ T cells assessed in each QVOA are reported in Table 4.

### Quantitation of SIV *gag* RNA.

Viral RNA was measured in plasma, CSF, cell culture supernatants, and tissues by reverse transcriptase (RT) qPCR (RT-qPCR) or RT-ddPCR as previously described (33, 34). In brief, viral RNA was isolated in duplicate from 140 μl of plasma or supernatant using a QIAamp viral RNA minikit (Qiagen, Valencia, CA, USA) according to the manufacturer’s protocol. For tissues, total RNA was isolated from 50 mg of tissue in singlet or triplicate using an RNeasy kit (Qiagen) according to the manufacturer's protocol. As suggested by the manufacturer’s protocol, an on-column DNase digestion was performed for all samples using an RNase-free DNase set (Qiagen) and 3 units of RQ1 DNase (Promega, Madison WI). Quantification of SIV RNA was performed by RT-qPCR using a QuantiTect virus kit (Qiagen) or RT-ddPCR with a ddPCR Supermix for Probes kit (Bio-Rad) and a primer/probe set for SIV *gag*: primer SIV21F (5′-GTCTGCGTCATCTGGTGCATTC-3′), primer SIV22R (5′-CACTAGGTGTCTCTGCACTATCTGTTTTG-3′), and probe SIV23 (FAM-5′-CTTCCTCAGTGTGTTTCACTTTCTCTTCTG-3′-BHQ1, where FAM is 6-carboxyfluorescein and BHQ1 is black hole quencher 1) (Integrated DNA Technologies, Coralville, IA, USA). A Rotor-Gene Q thermocycler (Qiagen) was used for RT-qPCRs, and a QX-100 droplet digital PCR system (Bio-Rad) was used for ddPCRs, as previously described (86). Samples were assessed by RT-ddPCR when there were less than 10 copies/PCR mixture detected by RT-qPCR.

### Quantitation of SIV *gag* DNA.

DNA was isolated from tissues using an AllPrep DNA/RNA kit (Qiagen) according to the manufacturer’s recommendations. Viral DNA was measured in tissues by multiplex qPCR with a multiplex PCR kit (Qiagen) or ddPCR with a ddPCR Supermix for Probes kit (Bio-Rad) using primers in the SIV *gag* region and the macaque IFN-β gene for sample normalization and cellular quantitation. A Rotor-Gene Q thermocycler (Qiagen) was used for RT-qPCRs, and a QX-100 droplet digital PCR system (Bio-Rad) was used for ddPCRs, as previously described (53, 88). Samples were assessed by ddPCR when there were less than 10 copies detected by qPCR.

### Quantification of cellular SIV *tat*/*rev* RNA.

Quantification of SIV *tat*/*rev* RNA was performed by RT-qPCR using a QuantiTect virus kit (Qiagen) and a primer/probe set for SIV *tat*/*rev*: forward primer 3′-CGMARGAGAAGAAGAACTCCGAARAAG-5′, reverse primer 3′-CTATCTGYCAAGGCCARGA-5′, and probe FAM-5′-AACCAGAGAAGGMRAAGAAGGAGACGGTGM-3′-BHQ1 (Integrated DNA Technologies, Coralville, IA, USA). Three reactions were performed for each sample. To control for DNA contamination, one reaction mixture without reverse transcriptase was analyzed. Reaction mixtures were analyzed using a CFX96 real-time PCR detection system (Bio-Rad) with the following cycles: 50°C for 30 min, 95°C for 5 min, and 40 cycles of 95°C for 15 s, 54°C for 30 s, and 60°C for 1 min.

### Flow cytometry.

Isolated CD4^+^ T cell and monocyte purities were analyzed as previously described (33). In brief, the purities of cells from the spleen, lung, and PBMCs were assessed by comparing preselection suspensions, postselection CD11b^+^ cells, and postselection flowthrough. Samples were stained in 100 μl PBS–2% fetal bovine serum with mouse anti-human CD3-phycoerythrin (PE) (clone SP34-2; BD Biosciences, San Jose, CA), CD11b-fluorescein isothiocyanate (Beckman Coulter, USA), or CD4-Brilliant Violet 650 (clone OKT4; BioLegend, San Diego, CA) and LIVE/DEAD Aqua stain (Invitrogen). Whole blood was stained with CD3-PE (clone SP34-2; BD Biosciences, San Jose, CA), CD4-Brilliant Violet 650 (clone OKT4; BioLegend, San Diego, CA), and CD8-Brilliant Violet 570 (clone RPA-T8 BioLegend, San Diego, CA). Samples were incubated for 20 min at room temperature and fixed for 10 min with BD fluorescence-activated cell sorting lysing solution (BD Biosciences, San Jose, CA). Samples were acquired using a BD LSRFortessa flow cytometer (BD Biosciences). All data were analyzed using FlowJo software (FlowJo, Ashland, OR). The percentage of total CD3^+^ CD4^+^ CD8^−^ cells in whole blood was used to calculate CD3^+^ T cell contamination in monocyte/Mϕ cultures of the Mϕ QVOA (Table 3).

### *In vitro* infection of PBMCs.

Peripheral blood mononuclear cells (PBMCs) from uninfected pigtailed macaques were isolated by Percoll density gradient. Isolated PBMCs were cultured in 50 ml of RPMI medium supplemented with 10% fetal bovine serum, 1% penicillin-streptomycin (Gibco), 1% l-glutamine (Gibco), and 1% HEPES 1 M (Gibco) and stimulated for 48 h with 2 μg/ml recombinant human IL-2 (Life Technologies, Inc.) and 2 μg/ml phytohemagglutinin, M form (Life Technologies, Inc.). The activated PBMCs were then washed with Dulbecco’s phosphate-buffered saline (DPBS) and counted. Activated cells (1 × 10^6^) were spinoculated (2 h at 1,200 × g, room temperature) with 500 μl of supernatant from positive Mϕ QVOA wells from spleen, lung, and blood assays. After spinoculation, the cells were washed once with DPBS and resuspended in 1 ml of complete RPMI, plated in a 24-well plate, and incubated at 37°C for 14 days. Supernatants were collected at 5, 7, 10, and 14 days postinfection, and fresh medium was replaced at each time point. RNA was isolated from 1 ml of sample using a QIAamp MinElute virus vacuum kit (Qiagen), and SIV *gag* RNA was quantitated by RT-qPCR as described above.

### Statistics.

Infected cell frequencies in limiting dilution assays were calculated using the IUPMStats (v1.0) infection frequency calculator (http://silicianolab.johnshopkins.edu) (89).
